# Hamsters in the city: A study on the behaviour of a population of common hamsters (*Cricetus cricetus*) in urban environment

**DOI:** 10.1371/journal.pone.0225347

**Published:** 2019-11-21

**Authors:** Anna Flamand, Nancy Rebout, Camille Bordes, Lauréline Guinnefollau, Matthieu Bergès, Fanny Ajak, Carina Siutz, Eva Millesi, Christiane Weber, Odile Petit

**Affiliations:** 1 Laboratoire Image Ville Environnement, UMR 7362, Faculté de Géographie et de l’aménagement, Strasbourg, France; 2 Cognitive and Social Ethology Group, UMR Physiologie de la Reproduction et des Comportements, CNRS, IFCE, INRA, Université de Tours, Nouzilly, France; 3 Ecole Nationale Supérieure Agronomique de Toulouse, Castanet-Tolosan Cedex, France; 4 IPHC- DEPE, UMR 7178, Université de Strasbourg-CNRS, Strasbourg Cedex 2, France; 5 Université de Tours, Tours Cedex 1, France; 6 Department of Behavioural Biology, University of Vienna, Althanstrasse, Vienna, Austria; 7 TETIS CNRS, Université de Montpellier, Montpellier, France; University of Regina, CANADA

## Abstract

Animals in urban environments face challenging situations and have to cope with human activities. This study investigated the ecology and behaviour of a population of European hamsters (*Cricetus cricetus*) living in the city centre of Vienna (Austria). We recorded the surface activities of 35 hamsters in May 2015. Each focal animal was observed for 15 minutes, and a total of 66 focal samples were analysable. As a prey species in an environment teeming with human activities, we predicted a high level of vigilance by the hamsters. The results show that while animals dedicated a lot of time to vigilance, most of their time was spent foraging. The study also explores whether the frequency of vigilance behaviours differ between males and females. We found that vigilance behaviours were expressed in a different manner by males and females. Finally, we investigated the distribution of the burrows on green spaces depending on proximity to trees and on noise levels. We found a biased distribution of burrows, with a spatial preference for location protected by the vegetation and distant to noise sources. Although burrows were located preferentially under vegetation cover, levels of noise did not determine their positions. Moreover, this species does not respond to disturbances like daily urban noises, probably due to habituation. The common hamster is an endangered species; our results lead to a greater knowledge of its behaviour in a persistent urban population.

## Introduction

Urbanization is a growing phenomenon that has deep implications for ecosystems. One of them is the destruction of wildlife habitat, mainly in forest or agricultural areas [1,2]. When its habitat is destroyed, there are two possible solutions for an animal: moving to a more suitable environment or trying to face the new one [3]. In this latter situation, individuals have to adjust to urban conditions via a process called synantropism [4]. An often mentioned key element of successful adaptation in synantropic species is behavioural plasticity [5,6]. It could induce modifications like change in diet [5], adjustment of activity patterns according to human activities [7–9] or decreased levels of fear towards humans [10].

The common hamster (*Cricetus cricetus*) is one such synantropic species [11] that can be found on the outskirts or even in the centre of several large European cities [12,13]. It demonstrates some flexibility specific to urban context, which have been described above [14,15]. Nonetheless, this species–native to environments such as the steppes and grasslands–is usually found in agricultural areas of Europe and Asia [16]. The destruction of its natural habitat mainly caused by changes in agricultural practices and urban spread could explain its presence in towns [11,17]. Actually, some urban populations were already settled in these areas before the city expanded and they have adapted to their new environment.

The common hamster is a hibernating rodent, active from March to September [18]. Its active period is dedicated to reproduction and the storage of food in its burrow, in anticipation of hibernation. Over the previous decades and throughout the western part of the distribution area of the species, the populations of hamsters have dramatically declined [17]. Hence, the species is strictly protected by the Appendix IV of the Fauna-Flora-Habitat Directive in all countries of the European Union included in its distribution range (Austria, Belgium, Bulgaria, Czech Republic, Germany, France, Netherlands, Poland, Romania, Slovakia, and Slovenia) [19]. Several of these countries took some conservation measures including reintroduction programs, reinforcement of the national legislation and agricultural measures [17]. The species is recognized as an umbrella species which means that these protection measures would also provide benefits to other species and more broadly to biodiversity [20]. Despite extensive actions, the decline of rural populations is hard to counteract. The accommodation of the species to urban areas could be regarded as a chance to prevent the extinction process. It is thus of prime interest to study populations that have succeeded to adapt to an urban environment [21]. Some urban populations have been monitored for several years, providing information about settlement and distribution of populations as well as about reproduction and hibernation [11,16,22]. Nevertheless, knowledge is still missing on behaviour, an aspect addressed by few studies but not in depth [23,24]. Studies on behaviour–a crucial feature of adaptation–would provide a new and broader perspective on the species in urban areas. By gathering information on a free-ranging population living in urban areas, the ultimate objective of this study is to introduce hamsters into similar environments for conservation purposes as experienced in the Alister program (Life + Biodiversity, LIFE12-BIO-FR-000979) [25]. Characterizing beneficial and disruptive elements of the environment could help providing a suitable environment contributing to establish these urban populations.

For such purpose, the current study investigates the behaviour of an urban population of hamsters, and specifically considers how they allocate their time above ground. We focused on their activity budget (time spent for each activity at each trip). We assumed that they would be particularly vigilant and thus spend relatively short periods of time foraging given the inherent anthropic pressures of the urban environment. The range of vigilance behaviours expressed in response to this environment was also examined. Given the different reproductive strategies of males and female, males search for the burrows of females for mating purpose, while females need to forage given the high energetic demands of reproduction, the differences between males and females were analysed for both activity budget and vigilance behaviours. Additionally, the use of green spaces was also studied through the location of burrows depending on proximity to trees and on noise levels. As hamsters are prey animals, we expected a heterogeneous distribution with a spatial preference for location protected by vegetation and far from noise sources possibly perceived as a threat, preventing them to hear an approaching predator or to communicate.

## Material and methods

### Study site

**Fig 1:**
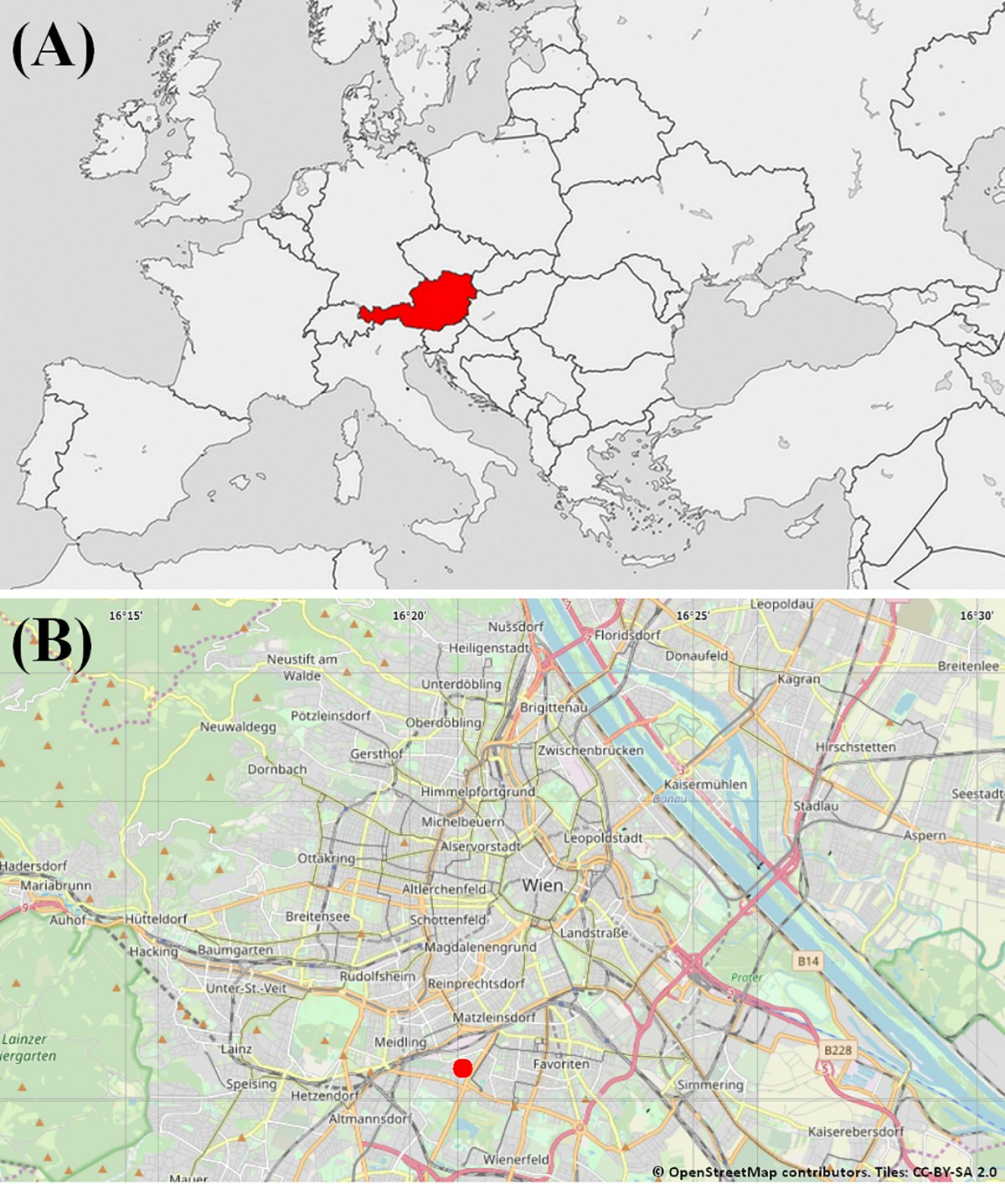
**Location of the study site in Europe (A) and in Vienna (B).** (A) Location of the country of study (B) Location of the study site in the city of Vienna.

The study area was located on the grounds of the Kaiser-Franz-Josef hospital in the South of the city of Vienna, Austria (geographic coordinates: 48°10'27.9"N 16°21'02.8"E, *cf*. Fig 1A and Fig 1B). This site is of interest because it has some classic characteristics of the urban environment (i.e. human presence and activities, lighting, traffic, etc.) and is not close to crops in which the species usually inhabits. This allows us to study how the common hamster deals with these urban elements without the presence of crops that provide food and protective vegetation cover. The results obtained in this study could constitute indications for allowing the implantation of hamsters in comparable sites for conservation purposes.

The study site covers 4.32 ha, including 2.85 ha of green areas (total surface) surrounding the hospital. These green spaces are mainly composed of grass with trees and bushes also bordering the plots. The hamsters were settled heterogeneously on these plots. The studied population of free-ranging hamsters was already present before the hospital was built in 1884. Since 2013, the University of Vienna monitored this population [22] which seems to cope with urban conditions, especially through its successful reproduction and usual hibernation pattern [26,27]. Despite slight fluctuations over the years, this population of about 170 individuals is considered stable.

### Procedure

Data were collected between 30 April and 26 May 2015, during the breeding season of the common hamster. We first estimated the number of burrows that were active i.e. inhabited by an individual, indicated by the presence of faeces, recent feeding food and/or recent excavated soil and inactive i.e. no longer used, indicated by the vegetation at the entrance and the absence of pathways around it. As it was impossible to ascertain which entrances were linked to a burrow system, every single entrance was defined as a burrow [28]. At the outset of the study, 685 burrows (194 active, 491 inactive) were detected. This number grew to 787 at the end of the study (208 active, 579 inactive). The spatial positions of these burrows were also recorded to evaluate whether their location was linked to the vicinity of trees or bushes and to the noise disturbances.

**Fig 2:**
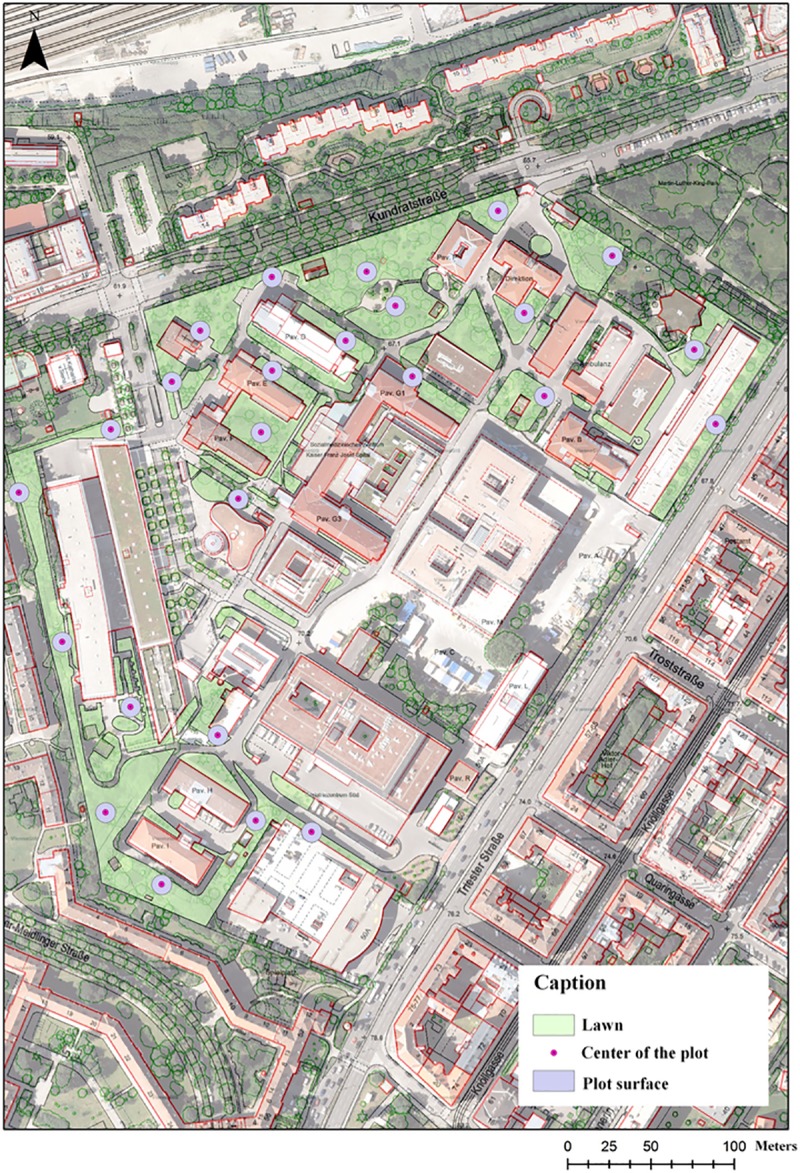
Detailed map of the studied site with plot positions.

To characterize the hamster habitat, 25 random points–hereafter referred to as plots–were randomly selected on the lawns. The plots were 25 metres apart, and each had a radius of 12 meters (*cf*. Fig 2). For each plot, the level of ambient noise was assessed with a sonometer (Testo, reference 815). The recorded noises were typical of urban environment (motorised vehicles, construction activities, pedestrians, etc.) [29]. Noise intensity was recorded daily at the beginning of each observation session, in the morning and in the evening.

Regarding proximity to a vegetation cover, a burrow was considered close if located at less than one metre from a tree or bush.

Secondly, the behaviour of 35 individuals (13 adult males, 18 adult females and 4 of unidentified sex) was monitored. Individuals were identified by fur-marking (see [30] for a detailed description of the method used). Observation sessions were performed from the same plots as those used for the characterisation of the habitat. The plot of each session randomly changed on a daily basis. Two observers in different locations recorded the behaviour of hamsters above ground with a video camera (Sony Handycam HDR-CX190E), observing from a distance of 6 to 20 meters to the animal. Each individual was observed using the focal sampling method [31] for a 15-minute period (the collected sequences are then referred as focal periods). If the focal individual disappeared from view, the observer stopped filming until it reappeared. As one animal could have been observed several times, the number of focal periods per animal has been nested by individual in the statistical analyses.

After five days of preliminary observations throughout the day and night, observation sessions alternated between 6 a.m. to 9 a.m. (morning session) one day, and 5 p.m. to 9 p.m. (evening session) the next day. These two periods correspond to the activity peaks reported in urban environments for this species [32]. Indeed, the common hamster is often described as a nocturnal species in the literature [33], yet it is also active at dusk [32] and even during the daytime [34]. Our observations, carried out at two different moments (morning and evening), allowed us to compare the repartition of activities (i.e. activity budget) and the frequency of vigilance behaviours according to the period during which the animals are active.

The observational protocols followed EU Directive 2010/63/EU guidelines for animal experiments. As this study does not include manipulations or direct interactions with the individuals studied, no specific permissions were required. However, the director of the hospital gave us authorization to observe the hamsters.

### Behavioural data collection

Overall, 66 focal periods (over a total of 156 hours of field work) were collected. After sex identification and the exclusion of poor-quality images, 54 focal periods remained. Video footage analysis was carried out according to the ethogram developed by Ziomek et al. (2009) [23], which was completed with our observations (*cf*. Table 1 for description of behaviours). During the observations, all behaviours were precisely recorded and for rare ones, regrouped in the category “other” (*cf*. Table 1) in the following statistical analysis.

**Table 1:** Ethogram (adapted from Ziomek et al. 2009).

Category	Subcategory	Elements of behaviour	Description
**Foraging**	Foraging (*per se*)		Collecting food using paws, transporting food to burrow
	Food sniffing		Progressing across the ground while scenting food
	Feeding		Gnawing and swallowing food, storing food in its cheek pouches
	Foraging/feeding		When feeding and foraging alternate too quickly to be considered as the only behaviour occuring
**Locomotion**	Horizontal locomotion		Moving, walking, running
	Vertical locomotion		Clinging, climbing, jumping
**Burrow**			The hamster is completely or partially inside its burrow.
**Vigilance**	Head-rearing		Head raised or standing erect on its hind legs, looking around.
	Monitoring		Standing erect on its hind legs, moving its head to scan its environment.
	Short-term freezing		Freezing for less than a second, stopping its activity and all its movements without scanning its environment.
	Long-term freezing		The same behaviour as short-term freezing, but lasting more than one second.
	Escape		Quickly running away from the disturbance.
**Social interactions**	Neutral	Social sniffing	Smelling in the direction of another individual, without physical contact.
		Direct identification	Nose-nose and nose-genital contact.
	Affiliative	Following	Travelling behind another individual.
		Play-fighting	Non-aggressive wrestling of juveniles
		Close	Two individuals remain less than 1m apart, with no physical contact.
		Hugging	Climbing onto another individual or flank contact.
	Agonistic	Social scent-marking	Rubbing its flank or urinating on the ground, leaving a scent.
		Approach	Coming close to an individual.
		Threat postures	Standing erect on its hind legs with the front legs stretched forward.
		Submissive postures	Lying on its back, exposing its ventral side or being press to the ground by another individual.
		Avoidance	Any movement preventing an interaction: jumping aside, running, walking away, etc.
		Intimidation	Running towards another individual without attacking.
		Attack	Chasing, biting, jumping upon other individual from behind or from the front.
		Defence	Boxing.
		Flight	Running away after a lost fight.
	Vocal	Vocalization	Squeaking, screeching, barking.
**Other**	Maintenance	Self-grooming	Scratching and grooming movements (cleaning a part or the whole body).
		Elimination	Urinating or defecating.
		Digging	Digging with the forepaws, sweeping away the earth with the hind paws, and pushing the earth aside with its rump.
		Scent-marking	Rubbing its ventral glands on the ground and leaving a scent.
		Sniffing	Smelling the ground near a burrow entrance (< 30 cm) without collecting food.
		No visible action	The hamster is out of view, or the action cannot be described.
		Not active	Motionless (inactive or sleeping).

### Statistical analysis

We performed statistical analyses using R 3.5.2 [35] and significance was set at p < 0.05.

We first explored the distribution of burrows according to the proximity to trees and bushes in order to know if this parameter had an influence on the location of the burrows. A chi-squared test was used to compare the number of burrows found in the centre of the lawns (without any cover protection) with the number of burrows located near trees. We subsequently investigated whether the number of burrows on each plot was dependent on noise intensity. To this end, we ran a generalized linear model (GLM) analysis in which the response variable was the number of burrows and the explicative variable was the noise intensity. Because the number of burrows was a count variable, we therefore used a Poisson distribution. The dredge function of the *“MuMIn”* package was used to select the best-fitting models, based on corrected Akaike Information Criterion (AICc), delta AIC (ΔAIC) and the Akaike weight [36] (S1 Table).

Analyses on activity budget of individuals were then conducted: at each surface trip of a hamster, the time spent for each activity was measured, giving a ratio of the different activities per trip. These different activity ratios were then compared according to sex (male or female) and time of day (morning or evening). These analyses were performed on 31 adult individuals among 35, since it was impossible to determine the sex of four focal individuals from the data and videos. We ran generalized linear mixed model (GLMM) analyses using the “*lme4”* package [37]. These were GLMMs of proportion in which success was considered to be the time spent on one activity and failure was the time spent on other activities. We therefore used a binomial distribution and a logit link function. The target variable was the proportion of time attributed to a given activity during the active period, and fixed factors were: type of activity (foraging, vigilance, social interactions, locomotion, burrow and other), sex and time of day. The target variable was expressed as proportions to allow for differences in video durations. In order to take into account the repeatability of individuals according to the observation sessions, individuals were considered as random factors and observation sessions were nested in individuals. After selecting suitable predictors (detailed above as fixed factors), we checked for the absence of correlation between these predictors using variance inflation factor (VIF). In each model, all VIF values were lower than 3. The dredge function of the *“MuMIn”* package was also used for model selection. We chose the model with the lowest AICc [38] which led us to exclude the time of day of the analysis (S2 Table). Post-hoc tests were performed with the “*emmeans*” package [39] to investigate the results of interactions between qualitative variables.

The same procedure was followed for vigilance behaviours analyses: at each surface trip of a hamster, the frequency of specific vigilance behaviours was measured, giving a ratio of specific vigilance behaviours on the total vigilance behaviours expressed during a surface trip. We compared the ratios of these different vigilance behaviours between type of vigilance behaviour (e.g. short-term freezing, long-term freezing, head-rearing or monitoring) and sex, and between type of vigilance and time of day. Our objective was to explore if the different type of vigilance behaviour differed according to sex or time of day. A GLMM analysis was performed, using the same approach as described above for the activity budget but with the frequency proportion of each vigilance as target variable, and type of activity, sex and time of day as fixed factors. The model selected led us to exclude the time of day of the analysis (S3 Table).

## Results

### Location of burrows

The influence of the proximity of trees and bushes and of noise level on the location of the burrow has been analysed. Burrows were not equally distributed (chi-squared test: 6.98, df = 1, p<0.01). They were more often located near trees and bushes (n = 429, i.e. 55% of the burrows) rather than in the middle of the lawns (n = 355, i.e. 45% of the burrows). Moreover, noise intensity had no effect on the distribution of burrows across the different plots (estimate ± SD: -0.060 ± 0.045, z = -1.32, p = 0.186).

### Activity budget

Activity time ratios were compared between activity and sex (N = 31, n = 53 with N the number of focal animals and n the number of sessions used for comparison). The best-fitting model included activity, sex and the interaction between activity and sex (S2 Table: model 1). Post-hoc comparisons of the interaction between activity and sex revealed that females spent more time foraging than displaying vigilance behaviours (0.588 ± 0.031 *vs* 0.116 ± 0.02, z = 10.24, p <0.001). The proportion of time spent foraging was also higher than the time spent in social interactions (0.588 ± 0.031 *vs* 0.0032 ± 0.004, z = 5.45, p <0.001), in locomotion (0.588 ± 0.031 *vs* 0.036 ± 0.012, z = 10.13, p <0.001), in burrow (0.588 ± 0.031 *vs* 0.232 ± 0.026, z = 7.97, p <0.001) and in the “other” category (0.588 ± 0.031 *vs* 0.004 ± 0.003, z = 5.79, p <0.001). The proportion of time dedicated to vigilance behaviours was higher than that observed for social interactions (0.116± 0.02 *vs* 0.0032 ± 0.004, z = -3.29, p = 0.013), locomotion (0.116 ± 0.02 *vs* 0.036 ± 0.012, z = -3.25, p = 0.015) and in the “other” category (0.116 ± 0.02 *vs*. 0.004 ± 0.003, z = -3.42, p = 0.008). The proportion of time spent in the burrow was higher than in vigilance (0.232 ± 0.026 *vs* 0.116 ± 0.02, z = 3.39, p = 0.009), in locomotion (0.232 ± 0.026 *vs* 0.036 ± 0.012, z = -5.7, p <0.001), in social interactions (0.232 ± 0.026 *vs* 0.0032 ± 0.004, z = -4.06, p<0.001), and in the “other” category (0.232 ± 0.026 *vs* 0.004 ± 0.003, z = -4.26, p<0.001). No other comparisons yielded any significant difference.

Males spent significantly more time foraging than interacting socially (0.299 ± 0.043 *vs* 0.018 ± 0.013, z = 4.33, p <0.001) or in locomotion (0.299 ± 0.043 *vs* 0.044 ± 0.019, z = 4.48, p <0.001). More time was dedicated to vigilance than to social interactions (0.234 ± 0.04 *vs* 0.018 ± 0.013, z = -3.82, p = 0.002), locomotion (0.234 ± 0.04 *vs* 0.044 ± 0.019, z = -3.73, p = 0.003) and in the “other” category (0.234 ± 0.04 *vs* 0.032 ± 0.016, z = -3.86, p = 0.002). More time was spent in the burrow than in locomotion (0.274 ± 0.042 *vs* 0.044 ± 0.019, z = -4.22, p <0.001), in social interactions (0.274 ± 0.042 *vs* 0.018 ± 0.013, z = -4.15, p <0.001) and in the “other” category (0.274 ± 0.042 *vs* 0.032 ± 0.016, z = -4.28, p<0.001). We did not find any other significant contrast.

**Fig 3:**
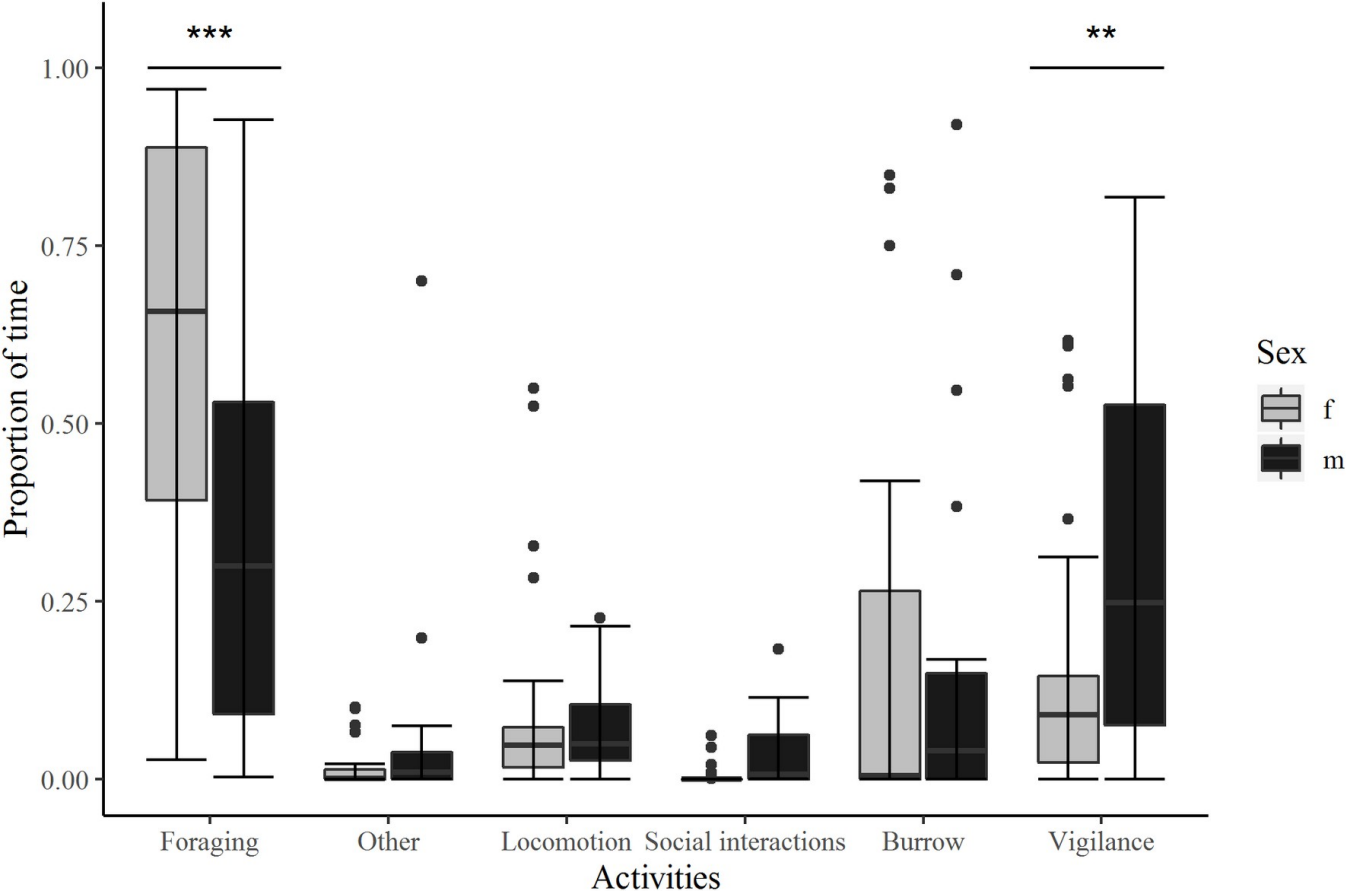
Activity budget of females and males. Proportion of time dedicated to the different behavioural activities (represented by the boxes), f: female; m: male. Estimate ± SD. **: p<0.01, ***: p<0.001. Outliers are symbolized by dots.

The comparison of male and female activity budgets (Fig 3) revealed that females spent significantly more time foraging than males (0.588 ± 0.031 *vs* 0.299 ± 0.043, z = 5.042; p <0.001) and that males spent more time displaying vigilance behaviours than females (0.232 ± 0.040 *vs* 0.116 ± 0.020, z = -2.82; p = 0.005). No statistically significant difference was found for other activities (female *vs* male: locomotion: 0.036 ± 0.012 *vs* 0.044 ± 0.019, z = -0.38; p = 0.71, social interactions: 0.0032 ± 0.004 *vs* 0.018 ± 0.013, z = -1.36; p = 0.18, burrow: 0.232 ± 0.026 *vs* 0.274 ± 0.042 z = -0.87; p = 0.38, others: 0.004 ± 0.003 *vs* 0.032 ± 0.016 z = -1.89; p = 0.059).

### Vigilance behaviours

Ratios of frequency of vigilance behaviours were compared between type of vigilance behaviour and sex (N = 30, n = 50 with N the number of focal animals and n the number of sessions used for comparison). The best-fitting model was explained by activity, sex and the interaction between activity and sex (S2 Table: model 1). Post-hoc analysis showed that females expressed a higher proportion of short-term freezing than head-rearing (0.41 ± 0.016 *vs* 0.31 ± 0.015, z = -4.86, p< 0.001). The proportion of short-term freezing was also higher than long-term freezing (0.41 ± 0.016 *vs* 0.24 ± 0.014 z = -7.96, p <0.001) and monitoring (0.41 ± 0.016 *vs* 0.037 ± 0.006, z = -15.97, p<0.001). Head-rearing was also more frequent than long-term freezing (0.31 ± 0.015 *vs* 0.24 ± 0.014, z = 3.20, p = 0.0075) and monitoring (0.31 ± 0.015 *vs* 0.037 ± 0.006, z = 13.31, p <0.001). The proportion of long-term freezing was higher than monitoring (0.24 ± 0.014 *vs* 0.037 ± 0.006, z = 11.40, p <0.001).

In males, the proportion of head-rearing was higher than long-term freezing (0.49 ± 0.020 *vs* 0.22 ± 0.017, z = 9.41, p <0.001), short-term freezing (0.49 ± 0.022 *vs* 0.22 ± 0.017, z = 8.72, p <0.001) and monitoring (0.49 ± 0.022 *vs* 0.065 ± 0.010, z = 9.53, p <0.001). Long-term freezing and short-term freezing were equally expressed (0.22 ± 0.017 *vs* 0.22 ± 0.017, z = 0.14, p = 0.99), but long-term freezing was more frequently expressed than monitoring (0.22 ± 0.017 *vs* 0.065 ± 0.010, z = 7.38, p <0.001). Short-term freezing was also more frequently expressed than monitoring (0.22 ± 0.017 *vs* 0.065 ± 0.010, z = -7.27, p <0.0001).

**Fig 4:**
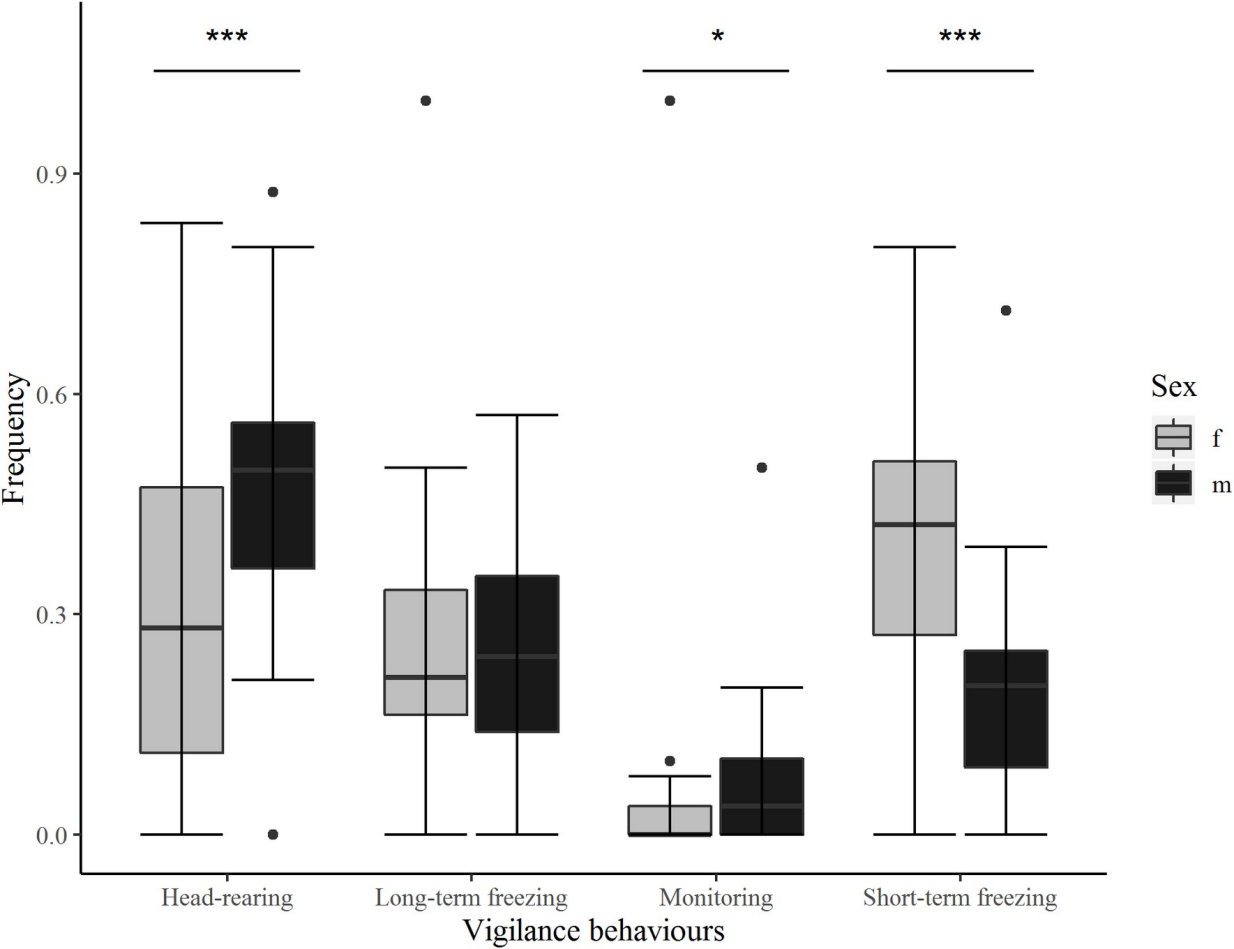
Frequency of vigilance behaviours in females and males. Proportion of frequency of vigilance behaviours (represented by the boxes), f: female; m: male. Estimate ± SD. *: p<0.05, ***: p<0.001. Outliers are reprezented by dots.

Comparison between sexes showed that females expressed more short-term freezing than males (0.41 ± 0.016 *vs* 0.22 ± 0.017, z = 7.71, p <0.001) and conversely, males expressed more head-rearing (0.49 ± 0.020 *vs* 0.31 ± 0.015, z = -7.20, p <0.001) and monitoring (0.065 ± 0.010 *vs* 0.037 ± 0.006, z = -2.50, p = 0.0124) than females (Fig 4). No intersex differences were found for long-term freezing (female *vs* male: 0.24 ± 0.014 *vs* 0.22 ± 0.017, z = 0.82, p = 0.41).

## Discussion

### Active period in urban areas

#### Location of burrows

Burrows were more frequently located near trees and bushes than in open ground. This distribution has already been described several times in urban areas [13,40], and the highest burrow density in towns has been observed close to bushes [11]. Such location choices can be explained by the protection the vegetation provides against predators, and the availability of food near trees and bushes. Indeed, leaves, seeds and fruits make up a significant proportion of the urban hamster diet [41], and are almost unlimited thanks to the maintenance of plants by gardeners [40]. Moreover, the roots could also offer useful support for digging burrows and lead to more stable burrows. Interestingly, the benefits provided by this location seem to overcome the disadvantages of the proximity or level of noise disturbances, suggesting that hamsters dig their burrows regardless of this pressure. A large number of the disturbances recorded were urban ambient noises. In different bird species, urban noises are problematic because of the value of the acoustic channel in social communication [42]. In contrast to birds, hamsters do not seem to be sensitive to this particular urban feature [40]. The common hamster is a solitary species for which social interactions are rare [23]. The limited use of vocal communication in hamsters could explain their tolerance of, and/or indifference to, ambient noises [11,40,43]. This result is consistent with reports of burrows located along main streets that are high-traffic roads in Simferopol, Ukraine [11,16]. Moreover, the studied population has lived near this hospital for many generations and is probably accustomed to these repetitive disturbances [44], which are similar to a background noise, whatever their intensity. Francis and Barber (2013) [45] suggested the possibility of a long-term decrease in sensory capacities in urban species, which could also explain why this stable urban population no longer reacts to urban noises. To sum up, the vegetal criteria—providing food source, protection and favourable soil features- seems to have an importance in location of burrows in urban environment, contrarily to noise levels. In the interest of the species, this information could be useful for the management of the green spaces: adding as many fruit trees and bushes as possible in places where hamsters are settled could help ensure the success of their reintroduction in urban areas [46].

#### Foraging

Our results show that foraging is the main activity in both male and female during their daily activity peaks, and this finding is in accordance with the species’ activity budget. Further, Hędrzak [24] demonstrate that suburban hamsters increase the return rate to their burrow and Ziomek et al. (2009) [23] found that hamsters tend to feed more inside their burrow than at the surface. This is in accordance with our results, which reveal that hamsters spend a large proportion of time inside their burrow during their activity period. This could also be explained by the proximity of certain food sources in the urban environment, allowing hamsters to consume their food directly inside their burrows. Thus, hamsters may forage and feed in a fractioned manner, with frequent round trips to their burrow. Such adjustment might allow hamsters to decrease the time exposed to threats outside their burrows and consume the food in a safety place.

#### Vigilance

While foraging is the main activity of hamsters, we found that they also dedicate an important part of their time to vigilance. As a consequence, their daily foraging is often interrupted by these behaviours, which might engender energetic costs. For prey species, vigilance is a costly but fundamental antipredator behaviour that affects survival. Although the urban environment decreases the diversity of the common hamster’s natural predator species [11,16], mortality due to predation still exists [46]. The occurrence of vigilance could be due to predation risk, but it could also reflect a reaction to anthropogenic disturbances [5]. Indeed, hamsters that live in an urban environment are considered more cautious and vigilant than their rural counterparts [24,40].

#### Trade-off foraging-vigilance: Two different sex strategies

Like many prey species, hamsters have to balance between foraging and vigilance [47] leading to different strategies in males and females during the breeding season. Indeed, we found that females spent more time foraging than males, a result that Feoktistova et al. (2014) [16] also found in hamsters later in the breeding season. This result is not surprising because females have high energy needs during the breeding period due to the reproduction costs of gestation and lactation [48]. The high availability of food in urban areas causes an early start of the breeding season [46] and lengthens its duration [7], entailing a particularly extended effort for females [26]. In addition, males change burrows frequently during the mating period when searching for females, and therefore probably store less food in their temporary burrow, leading to less foraging.

On the other hand, males dedicate more time to vigilance than females do. Sexual competition may lead males to increase their vigilance in order to detect the presence of potential competitors and/or avoid aggressive encounters [49].

Moreover, the different vigilance behaviours have variable energetic costs, and this could influence their relative expression in males and females. Among vigilance behaviours, males regularly express head-rearing and do so more often than females. This behaviour provides information about the environment and causes a short interruption of activities. It could even be expressed during foraging/feeding [50] reducing the cost of vigilance to some extent.

Females expressed more short-term freezing than males. Conversely to head-rearing, freezing (short or long) is an exclusive behaviour that implies a longer and complete interruption of the animal’s current activity. The hamster obtains limited information about the environment with this kind of behaviour, but its stillness has the advantage of not requiring any physical effort. Indeed, females focus their efforts on gestation and maternal care during the breeding season, so all other energetic costs due to spatial displacements could be restricted. Females may therefore choose a vigilance strategy that requires the least possible energetic effort. In contrast to males, their stationary behaviour could explain that they invest less time in vigilance behaviours.

## Conclusion

In this urban population, hamsters select the location of their burrow in order to gain maximum benefits from their habitat. Moreover, the trade-off between foraging and vigilance differs depending on sex, potentially due to reproductive strategies. Although the common hamster does not seem to be influenced by the noise disturbances when selecting the location of its burrow, like other wildlife species are, it faces new stressors in urban conditions (i.e. human proximity, road traffic, light or soil pollution, etc.). The consequences of the resulting biological constraints [7] should be carefully monitored to ensure the conservation of this endangered species in urban settings for already existing populations. Additionally, the destruction of the natural habitat of the common hamster is constantly expanding everywhere in Western Europe, which has severe consequences on its conservation status. The results of the current study might be applied to arrange urban areas in order to render them suitable for reintroduction purposes of the species.

## Supporting information

S1 Table GLMM models selection table for the analysis of location of burrow according to noise intensity. Models ranked by the Akaike Information Criterion (AICc): AICc computations and relative variable importance (weight) are indicated for each model. The best model is represented in bold.
(DOCX)

S2 Table GLMM models selection table for the analysis of the activity budget. Models ranked by the Akaike Information Criterion (AICc): presence (+) for qualitative effects, AICc computations and relative variable importance (weight) are indicated for each model. The best model is represented in bold.
(DOCX)

S3 Table GLMM models selection table for the analysis of the vigilance behaviours. Models ranked by the Akaike Information Criterion (AICc): presence (+) for qualitative effects, and AICc computations and relative variable importance (weight) are indicated for each model. The best model is represented in bold.
(DOCX)

S1 File Data file for the analysis of number of burrows according to noise intensity. 1^st^ raw: Letter corresponding to the plot in the green areas where burrows are located, 2^nd^ raw: number of active burrows found in each specific plot, 3^rd^ raw: Mean of the noise intensity per plot (in dB).
(CSV)

S2 File Proportion of time dedicated to the different behavioural activities in females and males. 1^st^ raw: Name of the observation session, 2^nd^ raw: Time of the day (Morning or Evening), 3^rd^ raw: Letter corresponding to the focal individual followed, 4^th^ raw: Sex of the focal individual (female (f) or male (m)), 5^th^ raw: Name of the activity performed by the focal individual (foraging, burrow, locomotion, others, social or vigilance), 6^th^ raw: Duration of the activity of the focal individual, 7^th^ raw: Total duration of the observed trip of the focal individual.
(CSV)

S3 File Frequency of vigilance behaviours in females and males. 1^st^ raw: Name of the observation session, 2^nd^ raw: Time of the day (Morning or Evening), 3^rd^ raw: Letter corresponding to the focal individual followed, 4^th^ raw: Sex of the focal individual (female (f) or male (m)), 5^th^ raw: Name of the specific vigilance behaviour expressed by the focal individual (Head-rearing, monitoring, long-freezing or short-freezing), 6^th^ raw: Frequency of the specific vigilance behaviour of the focal individual, 7^th^ raw: Total frequency of the vigilance behaviours for a focal individual.
(CSV)
