# Supplementary material for: Hamsters in the city: A study on the behaviour of a population of common hamsters (Cricetus cricetus) in urban environment
Source: PLoS One. 2019 Nov 21;14(11):e0225347. doi: 10.1371/journal.pone.0225347 (PMC6872164; doi:10.1371/journal.pone.0225347)
Supplement: S1 Table — Models ranked by the Akaike Information Criterion (AICc): AICc computations and relative variable importance (weight) are indicated for each model. The best model is represented in bold. (DOCX) [file pone.0225347.s001.docx]

| Model | Intercept | Df | logLink | AICc | ∆ AICc | Weight |
| --- | --- | --- | --- | --- | --- | --- |
| 1 | 0.5878 | 1 | -46.772 | 95.7 | 0.00 | 0.56 |
| 2 | 3.6700 | 2 | -45.806 | 96.2 | 0.44 | 0.45 |

**S1 Table : GLMM models selection table for the analysis of location of burrow according to noise intensity.** Models ranked by the Akaike Information Criterion (AICc): AICc computations are indicated for each model.
