# Supplementary material for: Hamsters in the city: A study on the behaviour of a population of common hamsters (Cricetus cricetus) in urban environment
Source: PLoS One. 2019 Nov 21;14(11):e0225347. doi: 10.1371/journal.pone.0225347 (PMC6872164; doi:10.1371/journal.pone.0225347)
Supplement: S3 Table — Models ranked by the Akaike Information Criterion (AICc): presence (+) for qualitative effects, and AICc computations and relative variable importance (weight) are indicated for each model. The best model is represented in bold. (DOCX) [file pone.0225347.s003.docx]

| Model | Activity | Moment | Sex | Activity :Moment | Activity :Sex | Intercept | df | logLink | AICc | ∆ AICc | Weight |
| --- | --- | --- | --- | --- | --- | --- | --- | --- | --- | --- | --- |
| **1** | **+** |  | **+** |  | **+** | **-0.8132** | **10** | **-543.631** | **1108.4** | **0.00** | **0.66** |
| 2 | + | + | + |  | + | -0.8132 | 11 | -543.631 | 1110.7 | 2.24 | 0.22 |

**S3 Table: GLMM models selection table for the analysis of the vigilance behaviours**. Models ranked by the Akaike Information Criterion (AICc): presence (+) for qualitative effects and AICc computations are indicated for each model. The best model is represented in bold.
